# Clinician-deployable deep hypergraph model integrating clinical and CT radiomics predicts immunotherapy outcomes in NSCLC

**DOI:** 10.1371/journal.pdig.0001361

**Published:** 2026-04-20

**Authors:** Jiangdian Song, Qiang Nie, Siqi Wang, Taixue An, Xiao Li, Yinlong Liu, Hairui Wang, Ruichuan Shi, Lu Wang, Meiyin Lin, Xuanda Pan, Xin Li, Qiuyang Hou, Nan Xu, Xiaoyu Guo, Yuqiang Mao, Bin Liu, Xiujuan Qu, Jian-Hua Liu, Wen-Zhao Zhong

**Affiliations:** 1 School of Health Management, China Medical University, Shenyang, Liaoning, China; 2 Department of Pulmonary Surgery, Guangdong Lung Cancer Institute, Guangdong Provincial People’s Hospital (Guangdong Academy of Medical Sciences), Southern Medical University, Guangzhou, China; 3 Ganzhou Municipal Hospital, Ganzhou, Jiangxi, China; 4 Department of Laboratory Medicine, Nanfang Hospital, Southern Medical University, Guangzhou, China; 5 Faculty of Data Science, City University of Macau, MacauChina; 6 Department of Radiology, Shengjing hospital of China Medical University, Shenyang, Liaoning, China; 7 Department of Medical Oncology, Guangdong Provincial People’s Hospital, the First Hospital of China Medical University, Shenyang, Liaoning, China; 8 Shengjing Hospital of China Medical University, Shenyang, Liaoning, China; 9 Department of Oncology, Guangdong Provincial People’s Hospital (Guangdong Academy of Medical Sciences), Southern Medical University, Guangzhou, China; 10 Department of General Practice, The First Affiliated Hospital of Guangzhou Medical University, Guangzhou, China; 11 Department of Radiology, The First Affiliated Hospital of University of Science and Technology of China (USTC), Division of Life Sciences and Medicine, USTC, Hefei, Anhui, China; 12 Department of Thoracic Surgery, Shengjing hospital of China Medical University, Shenyang, Liaoning, China; 13 Department of Radiology, Guangdong Provincial People’s Hospital (Guangdong Academy of Medical Sciences), Southern Medical University, Guangzhou, China; Dana-Farber Cancer Institute, UNITED STATES OF AMERICA

## Abstract

Current image-based deep learning models that predict the benefits of immunotherapy in non-small cell lung cancer (NSCLC) require high-performance hardware. We aimed to develop and externally validate a clinician-operable prognostic model that integrates clinical and imaging data in a format usable by clinicians on standard central-processing-unit (CPU) hardware. This multicenter study included 1,379 patients with NSCLC treated with immunotherapy from five Chinese hospitals and the Memorial Sloan-Kettering (MSK) Cancer Center. A pairwise association encoder (PAE) converted routinely collected baseline clinical variables into edge weights of a patient-similarity graph, while radiomics features extracted from pretreatment chest computed tomography (CT) were embedded as node attributes in a Deep Hypergraph for NSCLC (DHGN). DHGN was trained on progression-free survival (PFS) and validated on overall survival (OS). Model performance was compared with three established control models and a published deep learning benchmark. Ten thoracic oncology experts independently implemented and tested each pipeline. Trained on PFS, DHGN yielded C-indices of 0.72, 0.71, and 0.71, and 0.70, 0.71, and 0.69 when validated on OS. DHGN outperformed the clinical-only, radiomics-only, composite, and EfficientNetV2 models for both endpoints (all *P* < 0.0001). It correctly identified patients likely to achieve PFS > 24 months and those unlikely to reach 12 months, which was superior to tumor mutation burden (TMB) and PD-L1 expression. Higher DHGN scores strongly predicted longer survival outcomes (hazard ratio: 0.10, 95% CI: 0.07–0.16, *P* < 0.0001) and improved the prognostic accuracy of patient stratification based on PD-L1 expression. Based on clinicians’ practical deployment, the DHGN significantly reduced the operational complexity and computational requirements (*P* < 0.01) for model development and application in clinical settings. In conclusion, we proposed a clinician-friendly population graph model that fuses baseline clinical data with CT radiomics on widely available CPU hardware accurately stratifies NSCLC patients for immunotherapy benefits, potentially redefining benchmarks for non-invasive prognostic biomarkers and enabling broader clinical translation.

## Author summary

In this study, we aimed to build a practical tool that helps doctors predict which patients with non-small cell lung cancer are more likely to benefit from immunotherapy. Most existing deep learning prediction tools rely on complex computer systems that require powerful hardware and are difficult to use in routine clinical practice. To address this, we developed a new method that combines information from standard medical records and chest CT scans taken before treatment using a computer model that can run on regular hospital computers. We tested this tool on data from over 1,300 patients across six hospitals, including one in the United States, and found that it could accurately identify patients who were likely to live longer with immunotherapy and those unlikely to benefit from the treatment. Our model also outperformed other commonly used methods, including those based solely on CT data or common genetic tests. Importantly, we asked oncologists to use our tool directly, and they reported that it was both easy to use and saved time. By making this type of personalized prediction more accessible, our work could help improve treatment planning and bring the benefits of precision medicine to a wider group of patients.

## Introduction

Non-small cell lung cancer (NSCLC) represents a leading cause of cancer-related mortality worldwide [1]. Beyond targeted therapies for patients with specific driver genes (such as EGFR, KRAS, and ALK mutations), immune checkpoint inhibitors have emerged as a pivotal treatment strategy, significantly improving survival outcomes in patients with NSCLC [2–5]. However, current biomarkers, such as PD-L1 expression and tumor mutation burden (TMB), face limitations related to accuracy, tissue availability, tumor heterogeneity, and clinical cost [6–8]. This underscores an urgent need for robust prognostic tools for NSCLC [9].

Pre-therapy computed tomography (CT) image-based deep learning methods have shown promise for predicting immunotherapy outcomes in NSCLC; however, several challenges impede their clinical translation [10–12]. Although deep learning-based prognostic signatures have been externally validated [11,13–15], their interpretability and reproducibility require further evaluation. In addition, most such models are designed in academic and engineering laboratories, where high-performance computing resources such as graphics processing units (GPUs) are routinely available. However, when considering the development and deployment of such AI models in broader clinical settings, cost-effectiveness becomes a critical factor because investments in computational infrastructure and associated maintenance have substantial implications for healthcare institutions [16–18]. Recent immunotherapy prediction models, such as LORIS [19], integrate imaging features with clinical variables to provide a clinically accessible approach for outcome prediction. However, most existing deep learning frameworks treat clinical data as auxiliary inputs rather than fully integrating them into the computational architecture, thereby underutilizing the prognostic contribution of baseline clinical factors. Machine learning–based prognostic models, through risk-based regression methods (such as Cox or logistic models), incorporate clinical characteristics, laboratory findings, and other biomarkers into a unified framework that balances adaptability to routine clinical hardware with flexibility for multimodal data integration. In contrast, current deep learning approaches remain limited in their compatibility with CPU-based systems and their ability to incorporate structured clinical information, constraining their broader clinical translation.

Emerging graph convolutional networks (GCNs) have extended the representation capabilities of conventional convolutional neural networks to include irregular graphical data, thus offering a novel solution to these challenges [20]. By integrating clinical patient characteristics with imaging features in a population graph framework, GCNs facilitate the execution of deep learning computations on cost-effective hardware, thereby reducing dependence on expensive GPU-centric systems [21]. Recent studies have highlighted the potential of population-based GCNs for disease prediction and subtype classification [22,23], thus suggesting their potential for elucidating the interplay between baseline clinical characteristics and pretreatment CT images survival prediction for patients with NSCLC receiving immunotherapy.

In this study, we developed and validated a population graph model that integrates clinical features with CT imaging data to improve pre-therapy survival prediction in patients with NSCLC undergoing immunotherapy. Our approach combined CT imaging features with patient similarity measurements, offering a practical framework for deep learning models that are both clinically and translationally accessible.

## Methods

### Ethics statement

The study received ethical approval from the Institutional Review Board of Guangdong Provincial People’s Hospital (KY2025-473-02), and the First Affiliated Hospital of China Medical University (AF-SOP-07-1), and the First Affiliated Hospital of University of Science and Technology of China (2023-RE-113), and China Medical University (2025–126). This study was conducted in accordance with the 1964 Declaration of Helsinki. Owing to the retrospective design of the study, the requirement for informed consent was waived.

### Study design

In this multicenter cohort study, we developed a *Deep Hypergraph* model for NSCLC (DHGN) to predict immunotherapy survival outcomes using routinely available CT images and baseline clinical data. We used a two-step patient-to-graph approach, in which each patient was represented as a node in a population graph, and their baseline characteristics were encoded using a pairwise association encoder (PAE) to quantify clinical similarity. The DHGN model, built upon Hypergraph Neural Networks [24], was then applied to this graph, incorporating pre-therapy CT imaging features as node attributes. This integrative framework mirrors the actual clinical evaluation process, wherein baseline clinical data are considered first by clinicians before CT images are assessed concurrently, thereby overcoming the limitations of conventional Cox methods that directly fuse multimodal data.

### Participants

**Fig 1:**
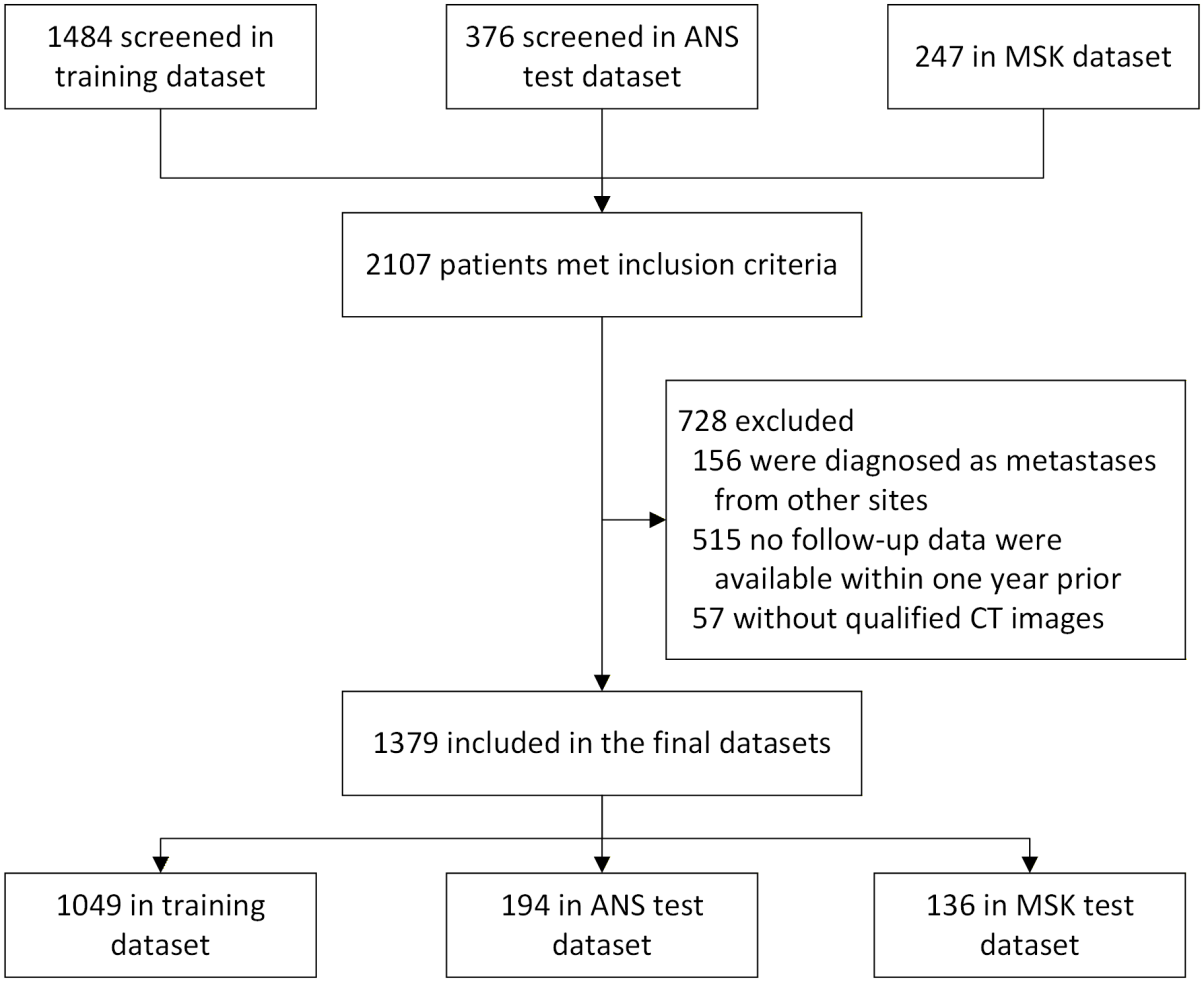
Flowchart of the patient recruitment process for the study. ANS: patients from Nanfang Hospital of Southern Medical University, the First Hospital of University of Science and Technology of China, and Shengjing Hospital of China Medical University. MSK: patients from the Memorial Sloan-Kettering Cancer Center (https://www.cbioportal.org/).

Data of patients with NSCLC were retrospectively retrieved from five Chinese hospitals (between January 1, 2014, and June 30, 2023), and the Memorial Sloan-Kettering (MSK) Cancer Center (https://www.cbioportal.org/) [25]. Our inclusion criteria were as follows: (1) pre-treatment CT images acquired within one month before initiating immunotherapy; (2) baseline clinical data suitable for graph construction (e.g., demographic, radiological, and clinicopathological information); (3) treatment with either immune monotherapy or a combination of immunotherapy and other therapies (chemotherapy, radiotherapy, targeted therapy, or anti-angiogenic therapy); and (4) patients with at least 12 months of follow-up for progression-free survival (PFS) and overall survival (OS). The exclusion criteria were as follows: (1) metastases from non-primary lung tumors and (2) inadequate CT image quality to identify the primary tumor, including artifacts that obscure the clarity of tumor boundaries, image noise that prevents reliable extraction of imaging features, and substantial metal artifacts that impair diagnostic interpretation. The PAE and DHGN models were trained using datasets from Guangdong Provincial People’s Hospital and the First Affiliated Hospital of China Medical University and externally tested using data from Nanfang Hospital of Southern Medical University, the First Affiliated Hospital of University of Science and Technology of China, and Shengjing Hospital of China Medical University (i.e., the ANS dataset; see Fig 1) and the MSK datasets. The division between the training and external test datasets followed an approximate 80:20 ratio, and data from each independent center were kept intact and assigned entirely to either the training or testing set.

The primary PFS endpoint was defined as the interval from the initiation of immunotherapy to disease progression or death. Progression was identified based on imaging evidence of tumor growth, new lesion development, or physician assessments. OS was defined as the time from initiation of immunotherapy until death. Patients who survived without disease progression or death were censored at the final follow-up.

### Procedures

**Fig 2:**
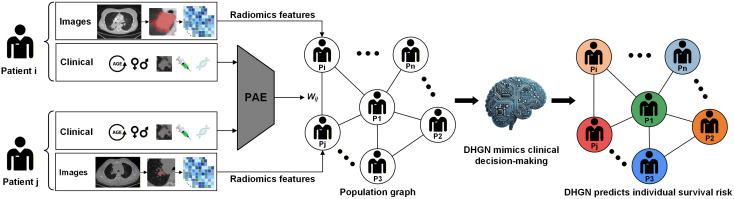
Flowchart of the study protocol. The proposed two-step strategy mimicked clinical diagnostic practice. First, the pairwise association encoder (PAE) computed similarities among the baseline characteristics of the patients to construct inter-node edges in the graph. The Deep Hypergraph architecture was then used to construct the Deep Hypergraph for NSCLC (DHGN) model by integrating these edges with pre-treatment computed tomography imaging features to enhance the prognosis of survival outcomes in patients with non-small-cell lung cancer undergoing immunotherapy.

The study workflow is illustrated in Fig 2. Nine baseline clinical characteristics were initially input into the PAE to quantify the pairwise associations between patients, with each patient comprising a node in the population graph. Local radiologists with >10 years of experience manually segmented the primary tumors on the CT images using ITK-Snap (version 3.6.0, http://www.itksnap.org) and extracted radiomics features to serve as node attributes. The radiomics features and PAE population graph were then used as DHGN inputs to predict survival outcomes. We also developed three control models concurrently: a clinical baseline characteristics-based Cox regression model, a radiomics feature-based Cox regression model, and a radiomics-clinical composite Cox regression model, as implemented in previous studies in this field. The performance of the DHGN was compared with that of these control models and an emerging deep learning model that used both GPU and CPU platforms. The DHGN’s prognostic accuracy was also validated against conventional biomarkers, including PD-L1 expression and blood-based TMB.

### PAE population graph

Nine baseline clinical characteristics: sex, age, smoking history (yes/no), pathological subtype (adenocarcinoma, squamous cell carcinoma, or other), morphology (solid, ground-glass nodule, or mixed), longest diameter, treatment line, combination therapy plan (combined therapy or monotherapy), and derived neutrophil-to-lymphocyte ratio, were used as PAE inputs to estimate inter-patient similarity. PAE consists of two parallel projection networks that share weights, followed by a cosine similarity measurement function. Clinical inputs for patient pairs ($P_{i}$, $P_{j}$) are first normalized and rescaled to unit-norm vectors to mitigate gradient vanishing during the training. These normalized vectors are then encoded by the parallel projection networks into hidden feature vectors $\;H_{i}$ and $H_{j}$ within a shared latent space. We used a cutoff value of 9.0 months (the median PFS in our datasets) to define the PAE label, with a label edge only being established between $P_{i}$ and $P_{j}$ if both patients’ PFS were either above or below the cutoff. The cosine similarity between $H_{i}$ and $H_{j}$ was then encoded by PAE and rescaled to the [0, 1] interval to quantitatively measure the strength of the connection between the patients.

### DHGN

Pre-treatment CT images were collected for radiomics feature extraction. Both plain and contrast-enhanced CT scans were included to improve the generalizability of the model. To mitigate bias, all segmentations were reviewed by a radiologist with >20 years of experience. Following the Image Biomarker Standardization Initiative (IBSI) guidelines and previous studies, MIRP was selected because it provides the highest IBSI-compliant feature reproducibility among the current radiomics software platforms [26,27]. In line with prior evidence that additional interpolation and discretization preprocessing can reduce radiomic feature reproducibility, no image preprocessing was performed to ensure full compliance with IBSI standards. Using the five predefined MIRP configurations, 1,221 radiomics features were extracted. Radiomics feature reproducibility was evaluated by two additional independent radiologists who repeated the process on a random sample of 100 patients according to the IBSI guidelines. The DHGN model was then developed to integrate the reproducible radiomic features with the PAE-derived population graph. It was trained to predict the PFS risk score and characterize inter-patient image-based associations, generating a prognostic graph to stratify survival risk. The architecture used the latest HGNN^+^ method for Deep Hypergraph [24], and for the initialization and optimization of detailed hyperparameters, please refer to the source code published in this study. In addition, a detailed explanation of the DHGN model is shown in S4 Fig and S5 Fig. After a five-fold cross-validation on the training set, the optimal model was applied to the two test datasets for external validation. In addition, once trained on PFS data, the model was locked and applied to OS prognostication to evaluate its performance in long-term survival.

### Comparative test

We constructed three control models based on the baseline clinical and radiomics data. A univariate Cox proportional hazards model was used to identify variables with significant prognostic value (*P* < 0.05). These were then incorporated into multivariate Cox models to construct separate clinical, radiomic, and combined clinical-radiomic control models. In addition, we constructed a DHGN-response model using objective response as the endpoint. Objective response was dichotomized into responders (CR and PR) and non-responders (SD and PD), and the model’s performance was compared with that of the latest LORIS model.

Sensitivity analyses were performed to evaluate the prognostic accuracy of the PAE models constructed using three, five, seven, and nine variables. We additionally assessed the DHGN model performance across clinically relevant patient subgroups, including different treatment strategies (monotherapy vs. combination immunotherapy and first-line therapy vs. other lines of therapy) and different imaging protocols (non-contrast CT and contrast-enhanced CT), to determine whether cohort differences introduced bias under varying clinical and imaging conditions.

To compare our supervised PAE approach with an unsupervised alternative, we constructed a Gower distance-based ε-radius patient similarity network. Gower distance integrates continuous, ordinal, and categorical clinical variables into a unified mixed-type distance metric [28,29]. Using a predefined ε-radius threshold, we generated a binary patient similarity network, assigning a value of 1 when the Gower distance between two patients fell within the ε-neighborhood and 0 otherwise.

### Clinical deployment

The DHGN model was deployed in a real-world clinical setting using easily available computing resources. A personal computer running the Windows 11 operating system (Microsoft, WA) on an 8-core CPU (Intel, CA) was deployed, and 10 clinical experts, including oncologists, radiologists, radiation technologists, and immunology laboratory specialists, were instructed on how to install the operating environment, as well as how to train and test the DHGN model. In parallel, we performed comparative analyses against the previously reported EfficientNetV2 deep learning model [30]. The EfficientNetV2 model was validated for its value for NSCLC immunotherapy prognostication using a server equipped with an RTX 4090 GPU (NVIDIA, Santa Clara, CA, USA) and Ubuntu operating system (version 18.04, https://ubuntu.com/). Clinical experts rated both models on a five-point Likert scale across nine indices: clinical availability, user-friendliness, environmental compatibility, operational complexity, time consumption, accuracy, clinical credibility, interpretability, and willingness to use. In terms of time requirements, lower scores indicated shorter processing times, whereas higher scores indicated greater favorability for the other indices.

### Statistical analyses

Control models were constructed using Cox proportional hazards regression. Kaplan-Meier survival analyses and log-rank tests were used to assess the significance of patient stratification across the models. Harrell’s concordance index (C-index) with a 95% confidence interval (CI) was calculated to evaluate the accuracy of survival predictions by comparing model-generated risk scores with actual outcomes. Continuous and categorical variables between datasets were compared using the Mann-Whitney U and Fisher’s exact tests. Sample size evaluation was performed using PASS (Version: 21.0.3; NCSS, LLC, East Kaysville, UT, USA) [31]. X-tile [32] was employed to determine the optimal cut-off values for categorizing DHGN scores into three groups (low, medium, and high) for comparison with the PD-L1 expression groups (<1%, 1–49%, and >49%). All tests were two-sided, with statistical significance set at *P* < 0.05, and were performed using R software (version 4.1.3; R Foundation for Statistical Computing, Vienna, Austria). The source code for the pairwise association encoder and Deep Hypergraph models, and the clinical baseline characteristics and radiomics features of the open-access dataset are available at https://github.com/JD910/DHGN. In addition, the corresponding CT images and manual segmentation masks are available at Mendeley Data: https://data.mendeley.com/datasets/cwtxww73sw/1.

## Results

### Patients

We initially collected data from 2,107 patients with NSCLC who received immunotherapy at the five participating Chinese hospitals and MSK Cancer Center. After applying the inclusion and exclusion criteria, 1,379 patients remained eligible for enrollment. The training dataset comprised 1,049 patients (89 censored), and the ANS and MSK test datasets included 194 (21 censored) and 136 (24 censored) patients, respectively (Fig 1). The mean patient age at immunotherapy initiation was 62.6 years (standard deviation: 9.4), and the median follow-up time was 40.0 months [interquartile range (IQR): 10.2–55.9]. Among them, 715 (51.8%) had adenocarcinoma, 1,048 (75.9%) were men, and 919 (66.6%) had a current or prior smoking history, while the median longest diameter was 41 mm (IQR: 30–66, Table 1).

**Table 1:** Demographics and clinical characteristics of the patients in the training, and two external test datasets (ANS and MSK).

	Training (n = 1,049)	ANS test (n = 194)	p value*	MSK test (n = 136)	p value†
Age	63 (56–69)	62 (56–68)	0.24	68 (61–73)	0.001
Sex			0.92		<0.0001
Male	835 (80%)	154 (79%)		59 (43%)	
Female	214 (20%)	40 (21%)		77 (57%)	
Histology			0.02		<0.0001
Adenocarcinoma	537 (51%)	79 (41%)		99 (73%)	
Squamous cell carcinoma	358 (34%)	83 (43%)		37 (27%)	
Other	154 (15%)	32 (16%)		0 (0%)	
Smoking			0.19		<0.0001
Yes	672 (64%)	134 (69%)		113 (83%)	
No	377 (36%)	60 (31%)		23 (17%)	
Morphology			0.05		0.71
Solid	880 (84%)	176 (90%)		118 (87%)	
Ground-glass nodule	15 (1%)	1 (1%)		1 (1%)	
Mixed	154 (15%)	17 (9%)		17 (12%)	
Longest diameter (mm)	42 (28–61)	57 (38–75)	<0.001	40 (24–66)	0.86
Treatment line			0.01		<0.0001
1	658 (63%)	151 (78%)		45 (33%)	
2	272 (26%)	30 (15%)		75 (55%)	
≥3	119 (11%)	13 (7%)		16 (12%)	
Clinical stage			0.10	NA	
I	47 (5%)	5 (3%)			
II	37 (3%)	11 (5%)			
III	430 (41%)	67 (35%)			
IV	535 (51%)	111 (57%)			
Therapy regimen			0.51		<0.0001
Monotherapy	82 (8%)	8 (4%)		130 (96%)	
Combination	967 (92%)	186 (96%)		6 (4%)	
dNLR	2.7 (1.7–3.9)	3.1 (1.9–4.7)	0.08	2.4 (1.7–3.8)	0.17
PD-L1 expression			0.01		<0.0001
<1%	164 (16%)	16 (8%)		61 (44%)	
1–49%	153 (15%)	38 (20%)		23 (17%)	
>49%	114 (11%)	19 (10%)		52 (39%)	
Tumor mutation burden (mut/Mb)	8.0 (3.9–15.0)	NA		7.5 (3.9–12.3)	0.35
Progression-free survival (m)	9.1 (4.3–14.6)	7.0 (3.2–14.9)	0.02	3.1 (1.6–9.5)	<0.0001
Overall survival (m)	25.6 (14.7–43.2)	18.1 (9.4–22.1)	0.003	5.2 (1.9–10.3)	<0.0001

Note: Data are presented as mean (SD), n (%), or median (IQR).

*Comparison between the training and ANS test dataset.

^†^Comparison between training and MSK test dataset.

NA: data unavailable.

### PAE and DHGN performance

Using PAE, we constructed 391,315, 15,151, and 6,719 graphical connections for the training, ANS, and MSK datasets, respectively. The PAE achieved a sensitivity/specificity of 91.3%/95.1% in the training dataset (five-fold cross-validation), 93.3%/89.5% in the ANS test dataset, and 91.6%/93.8% in the MSK test dataset. From the pre-therapy CT images, 1,221 radiomics features were initially extracted. Of these, 560 met the IBSI reproducibility criteria and were used as node attributes in the DHGN model. The DHGN model yielded an optimal prognostic performance after 100 training epochs. Notably, higher DHGN scores were correlated with improved survival outcomes. The model achieved C-indices (95% CI) for PFS of 0.72 (0.68–0.75) in the training dataset, 0.71 (0.67–0.75) in the ANS test dataset, and 0.71 (0.66–0.77) in the MSK test dataset. The corresponding performances for OS were 0.70 (0.65–0.75), 0.71 (0.66–0.76), and 0.69 (0.64–0.73), respectively.

### DHGN provides monotonic survival prediction

**Fig 3:**
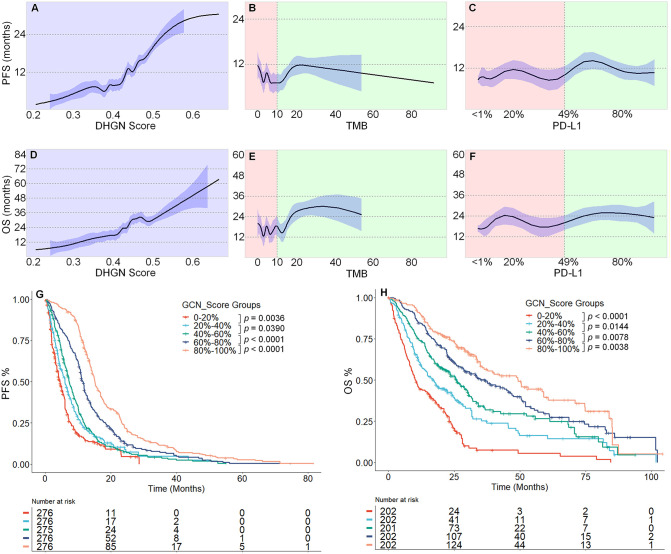
Relationship between DHGN, TMB, or PD-L1 and progression-free survival (PFS, A, B, and C, respectively) and overall survival (OS, D, E, and F, respectively). 95% CIs are shown using 1,000 bootstrap replicates. Regarding TMB and PD-L1, the vertical auxiliary lines indicate a TMB of 10 mut/Mb and PD-L1 expression of 49%. Kaplan–Meier analysis of DHGN on PFS (G) and OS (H) binned at the different percentiles. *P* values next to the legend indicate pairwise single-tail comparisons testing against the hypothesis that higher-scored patients have better survival than lower-scored patients.

We investigated the relationship between the participants’ DHGN scores and their outcomes. The results showed that as the DHGN increased, there was a consistent rise in PFS for participants, ranging from 0 to 32.8 months (95% quantile; Fig 3A). DHGN enabled the identification of participants who were highly likely to achieve a PFS > 24 months, while excluding those who were highly unlikely to achieve a PFS > 12 months (Fig 3). In contrast, neither TMB nor PD-L1 expression demonstrated adequate predictive power. No monotonic relationship was observed with PFS; neither high nor low TMB scores effectively distinguished between patients with prolonged or poor PFS, and PD-L1 expression showed a similar lack of predictive capability (Fig 3B and 3C).

We further observed that a higher DHGN score consistently predicted better OS among participants. Specifically, the hazard ratio between the lowest (0–10%) and highest (90–100%) DHGN groups was 9.8 (95% CI: 6.4–15.2, p = 1 × 10 ⁻ ²⁶). In contrast, no such monotonic relationship with OS was identified for TMB or PD-L1 biomarkers (Fig 3E and 3F). Stratification analysis showed that DHGN was strongly correlated with both short-term PFS and long-term OS when the 20% quantile was used to divide the patient groups (Fig 3G and 3H).

Sensitivity analyses demonstrated that the accuracy of the PAE model for estimating patient similarity improved as more baseline clinical variables were included. When nine variables were used, the PAE continued to significantly enhance the predictive performance of the DHGN model for immunotherapy outcomes (P < 0.001; S2 Table and S3 Table). In addition, subgroup analyses of factors potentially influencing model performance showed that treatment strategy was a potential influencing factor; the DHGN model achieved superior prognostic accuracy in patients receiving combination immunotherapy (the predominant treatment modality in our study) than in those receiving monotherapy. For PFS prediction, the C-index increased from 0.69 (95% CI: 0.64–0.74) to 0.75 (95% CI: 0.70–0.80) in the training set, and from 0.67 (95% CI: 0.63–0.71) to 0.72 (95% CI: 0.63–0.81) in the test set.

With respect to the imaging protocol, models constructed using contrast-enhanced CT showed clearly improved performance relative to those based on non-contrast CT. However, no obvious differences in prognostic performance were observed between first-line and non-first-line treatment subgroups. Detailed results are provided in S6 Table.

By searching for the optimal ε value in the Gower distance–based ε-radius patient similarity network, we identified ε = 0.5 as the optimal threshold. Using this unsupervised similarity measure, the DHGN model achieved a PFS C-index of 0.67 (95% CI: 0.61–0.73) in the training set, which was significantly lower than the PFS C-index of 0.72 (95% CI: 0.68–0.75) achieved using the 9-variable PAE-based DHGN model (P = 0.023). Similarly, significant performance differences were observed across both test sets (all P < 0.010). Furthermore, when the PFS-trained optimal models were fixed and evaluated for OS, the PAE-based DHGN consistently outperformed the unsupervised clustering approach (all P < 0.010).

### DHGN refines the decision-making of low-TMB and low-PD-L1 patients

**Fig 4:**
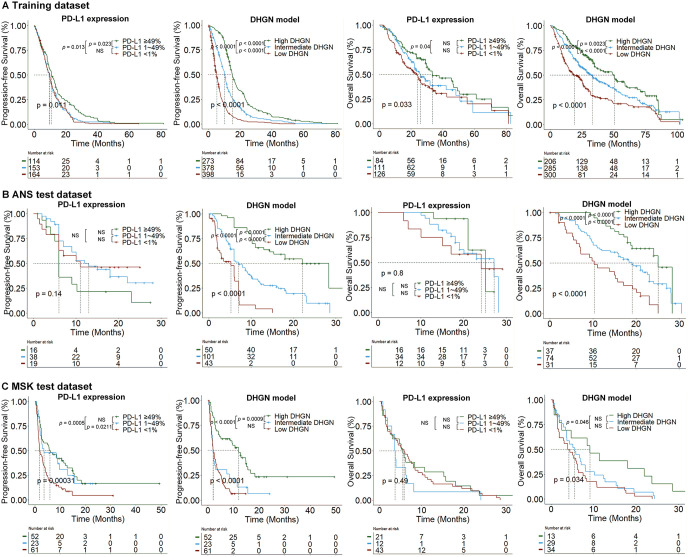
Kaplan-Meier survival curves for the training, ANS test, and MSK test datasets, after stratifying the DHGN model scores using the same proportions as the stratification by PD-L1 expression (38.0: 35.5: 26.5).

The results of this study showed that DHGN identified low-TMB and low-PD-L1 participants who could receive comparable survival benefits from immunotherapy to high-TMB and high-PD-L1 participants. In the comparative PD-L1 analysis, patients in the training dataset were stratified by PD-L1 expression into three groups: < 1% (38.0%, 164/431), 1–49% (35.5%, 153/431), and >49% (26.5%, 114/431), with corresponding median PFS (IQR) values of 9.1 (5.0–13.5), 9.4 (4.6–14.5), and 10.8 (5.4–17.1) months. When stratified by DHGN scores into low, intermediate, and high groups following the same ratio (38.0:35.5:26.5), the median PFS values were 4.6 (2.9–7.7), 10.1 (5.6–14.8), and 14.7 (11.2–22.8) months, respectively. The results showed that high-DHGN patients had a significantly better PFS vs. the PD-L1 > 49% group (*P* < 0.0001, log-rank test), while low-DHGN ones exhibited significantly poorer PFS vs. the PD-L1 < 1% group (*P* < 0.0001). Similar trends were observed for OS, with detailed comparisons between the test datasets (Table 2). Kaplan-Meier analyses in the training dataset demonstrated significant differences in both PFS and OS when patients were stratified by PD-L1 (*P* < 0.05) and DHGN (*P* < 0.0001, Fig 4A). However, in the test datasets, PD-L1 stratification yielded significant PFS differences only in the MSK dataset (*P* = 0.0003), whereas DHGN stratification produced significant differences in both PFS and OS in the ANS dataset (*P* < 0.0001 for both), and the MSK dataset (*P* < 0.05 for both, Fig 4C).

**Table 2:** Survival outcome comparison between PD-L1 expression and the Deep Hypergraph for NSCLC (DHGN) model score.

	PD-L1 < 1% (Ref)	Low DHGN score	P	PD-L1 1–49% (Ref)	Intermediate DHGN score	P	PD-L1 > 49% (Ref)	High DHGN score	P
Training (%)	38.0%	38.0%		35.5%	35.5%		26.5%	26.5%	
PFS	9.1 (5.0–13.5)	4.6 (2.9–7.7)	<0.0001	9.4 (4.6–14.5)	10.1 (5.6–14.8)	0.10	10.8 (5.4–17.1)	14.7 (11.2–22.8)	<0.0001
OS	18.0 (10.5–26.1)	15.8 (8.1–25.6)	0.03	21.1(12.4–27.6)	23.9 (14.5–38.5)	0.2	26.5 (15.5–34.3)	29.3 (19.7–46.1)	0.04
ANS test (%)	22.0%	22.0%		52.0%	52.0%		26.0%	26.0%	
PFS	6.0 (3.7–9.3)	3.7 (1.9–6.1)	0.02	10.0 (6.0–18.1)	6.0 (4.0–11.9)	0.01	10.0 (6.0–18.6)	17.6 (10.0–20.0)	0.01
OS	19.1 (13.8–24.6)	9.5 (3.7–17.5)	0.006	19.6 (17.1–23.8)	18.0 (9.6–21.7)	0.05	21.1 (18.1–22.6)	20.1 (17.8–23.3)	0.6
MSK test (%)	45.0%	45.0%		16.8%	16.8%		38.2%	38.2%	
PFS	1.9 (1.3–4.7)	2.1 (1.2–5.0)	0.7	3.6 (2.0–9.6)	2.2 (1.4–9.0)	0.2	5.9 (2.2–13.3)	9.5 (2.9–15.0)	0.02
OS	5.8 (1.7–10.5)	4.4 (1.5–7.7)	0.4	3.6 (2.6–6.3)	5.7 (2.8–10.7)	0.1	5.5 (1.9–15.3)	6.6 (2.5–16.3)	0.7

Note: % denotes the proportion of each PD-L1 expression patient group among the total patients in the dataset. DHGN score groups were divided according to the proportion of the PD-L1 expression groups (Ref). P value was calculated by log-rank test. PFS: progression-free survival, OS: overall survival.

For blood-based TMB, a previously validated cutoff of 10 mut/Mb was used [3,33]. Patients in the training dataset with TMB of ≥10 mut/Mb (41.9%, 117/280) had a median PFS of 8.9 months (IQR: 3.8–14.1) and median OS of 22.2 months (IQR: 12.5–31.3), while those with TMB of <10 mut/Mb (58.1%, 163/280) had a median PFS of 8.6 months (IQR: 5.2–12.6) and median OS of 16.1 months (IQR: 9.9–26.8). When using the analogous percentile (41.9:58.1) for DHGN binning, the highest 41.9^th^ percentile DHGN patients had significantly higher PFS (*P* < 0.0001) and OS (*P* = 0.002) than the TMB ≥ 10 mut/Mb group, and the lowest 58.1^th^ percentile DHGN ones had significantly reduced PFS compared with the TMB < 10 mut/Mb group (*P* = 0.01; Table 3). Kaplan-Meier analysis in the training dataset revealed significant differences in both PFS and OS with DHGN-based stratification (*P* < 0.0001; S1A Fig). In contrast, TMB-based stratification failed to differentiate survival outcomes (PFS: *P* = 0.25; OS: *P* = 0.11).

**Table 3:** Survival outcome comparison between tumor mutation burden (TMB) and the DHGN model score.

	TMB < 10 mut/Mb (Ref)	Low DHGN score	P	TMB ≥ 10 mut/Mb (Ref)	High DHGN score	P
Training (%)	58.1%	58.1%		41.9%	41.9%	
PFS	8.6 (5.2–12.6)	5.8 (3.2–10.2)	0.01	8.9 (3.8–14.1)	13.4 (9.8–20.7)	<0.0001
OS	16.1 (9.9–26.8)	18.5 (9.5–28.7)	0.9	22.2 (12.5–31.3)	29.0 (14.5–45.5)	0.002
MSK test	62.5%	62.5%		37.5%	37.5%	
PFS	2.6 (1.2–7.4)	2.1 (1.2–5.4)	0.08	4.7 (2.1–11.1)	9.5 (2.9–14.9)	0.01
OS	5.1 (1.6–9.1)	4.9 (1.7–9.5)	0.5	5.8 (2.7–16.3)	5.6 (2.8–10.6)	0.4

Note: % denotes the proportion of each TMB patient group among the total patients in the dataset. DHGN score groups were divided according to the proportion of the TMB groups (Ref). P value was calculated by log-rank test. PFS: progression-free survival, OS: overall survival.

Patients with high PD-L1 (>49%) accounted for 28.9% of the tested datasets, with a median PFS of 7.0 months. In contrast, an equivalent median PFS of 7.0 months was achieved for the highest 80.2^th^ percentile for DHGN binning. DHGN achieved an improvement of 51.3% in patients with NSCLC, demonstrating a similar survival benefit to high PD-L1 expression. Similarly, applying the highest 75.6^th^ percentile for DHGN binning yielded a median OS of 16.0 months, comparable to that of patients with high PD-L1 expression. Notably, this stratification identified an additional 46.7% of patients who derived long-term survival benefit.

**Fig 5:**
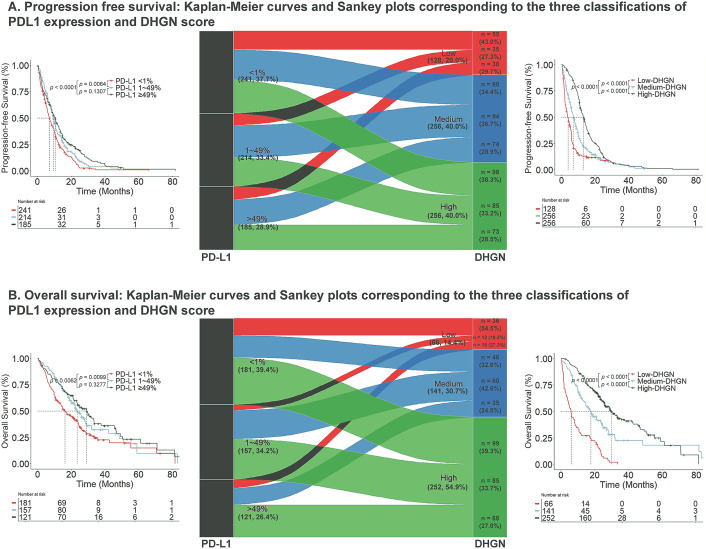
Sankey diagram tracks how patients initially stratified by PD-L1 expression (< 1%, 1–49%, > 49%) shift into low-, medium-, and high-risk groups defined by the X-tile–optimized DHGN score, and highlights the results change in progression-free survival (PFS) and overall survival (OS). A displays Kaplan–Meier curves for PFS under the PD-L1-based and DHGN-based three-tier classifications, while B shows the corresponding Kaplan–Meier curves for OS.

Using X-tile, we identified optimal DHGN score cut-off values 0.31 and 0.42 to define DHGN-Low, DHGN-Medium, and DHGN-High categories. Among patients with PD-L1 < 1%, 98 of 241 (40.7%) were reclassified into the DHGN-High group associated with better PFS; among those with PD-L1 1–49%, 85 of 214 (39.7%) were similarly reclassified (Fig 5). By incorporating these patients, the DHGN-High subgroup identified a substantially larger cohort of NSCLC patients for immunotherapy than using the PD-L1 > 49% criterion alone (185 vs 256 patients) and achieved comparable PFS (HR 0.89; 95% CI 0.72–1.10; *P* = 0.25). For OS, the corresponding reclassification rates were 99 of 181 (54.7%) for PD-L1 < 1% and 85 of 157 (54.1%) for PD-L1 1%–49%; therefore, the DHGN-High subgroup included 252 patients vs. 121 identified by PD-L1 > 49% alone and exhibited similar OS (HR 0.88; 95% CI 0.66–1.19; *P* = 0.40).

### Comparisons with control models

The three control models yielded C-indices ranging between 0.52 and 0.64 for PFS, and 0.40 and 0.62 for OS prognosis, across the training and test datasets (S1 Table). For both PFS and OS prognosis, the DHGN model significantly outperformed all control models (*P* < 0.01; S1 Table). In addition, in the training cohort, the DHGN-response model achieved an AUC of 0.71 (95% CI: 0.66–0.76). External validation in the ANS and MSK cohorts yielded AUCs of 0.71 (95% CI: 0.63–0.78) and 0.65 (95% CI: 0.61–0.69), respectively.

Subgroup analysis revealed that stratification by median DHGN score in patients with lung squamous cell carcinoma (Sqc) identified a significant survival benefit for high scorers, corresponding to median PFS and OS of 12.6 (IQR: 5.8–19.0) and 20.1 months (IQR: 12.7–24.1), respectively. Conversely, higher DHGN score patients with lung adenocarcinoma had a median PFS of 10.2 months (IQR: 5.0–16.7), which was not significantly different (*P* = 0.35); however, their OS median of 17.0 months (IQR: 6.8–20.0) was significantly lower than that of the Sqc group (*P* = 0.01). Among lower DHGN score patients, those with lung Sqc demonstrated significantly longer PFS compared to those with lung adenocarcinoma (3.8 [IQR: 1.7–6.6] vs 2.7 [IQR: 1.6–5.5] months; *P* = 0.004), although OS did not differ significantly (*P* = 0.65; S2 Fig).

### Clinical deployment

Using its recommended parameters [30], the EfficientNetV2 model achieved C-indices of 0.68, 0.67, and 0.60 for PFS in the training and two external test datasets, respectively, which were inferior to those of the DHGN model (*P* < 0.01). In addition, for the full dataset (n = 1,049 patients), both EfficientNetV2 and the proposed DHGN pipeline shared the same initial preprocessing requirement: approximately 300 CT slices per patient (512 × 512 resolution per slice) underwent segmentation-based preprocessing. Under identical GPU conditions, EfficientNetV2 required 12.60 min per training epoch and completed training after 100 epochs. Inference required 10 s per epoch, resulting in a total computational cost of 72.08 s per patient to generate prognostic predictions. For the DHGN pipeline, radiomics feature extraction required 25.26 s per patient. PAE training on the 1,049-patient dataset required 1.50 s per epoch (100 epochs total), and DHGN model training required 1.00 s per epoch (100 epochs in total). Overall, the per-patient computational cost of DHGN was 25.50 s. The whole DHGN pipeline remained 2.83 times more efficient than the advanced EfficientNetV2. In addition, EfficientNetV2 could not be deployed on CPU-only hardware owing to its GPU dependency, whereas the DHGN pipeline completed a full training epoch in only 3.50 s in a CPU-based environment. When including radiomics feature extraction, the total CPU deployment cost for DHGN was 32.63 s per patient.

Deployment assessments by 10 clinicians across diverse immunology-related specialties, using a five-point Likert scale (S3 Fig), revealed no significant differences in accuracy between the GPU-based EfficientNetV2 and CPU-based DHGN models. However, the DHGN model significantly outperformed EfficientNetV2 in terms of clinical availability, user-friendliness, environmental compatibility, operational complexity, time consumption, clinical credibility, interpretability, and willingness-to-use (*P* < 0.01).

## Discussion

This study demonstrates that a two-step population graph model could robustly predict immunotherapy outcomes in patients with NSCLC. By leveraging PAE to quantify clinical baseline similarities between patients and embedding radiomics features into a DHGN model, our approach achieved superior prognostic performance compared to tissue-based biomarkers and control models. This integrative strategy not only enhances the prediction of both PFS and OS, but also supports more refined patient survival risk stratification, thereby providing a promising tool for personalized immunotherapy decision-making and patient management.

One key strength of the proposed approach is its clinician-inspired design. In routine clinical practice, physicians first evaluate a patient’s baseline characteristics, such as age, smoking history, and blood test indicators, to form an initial assessment, with which they then incorporate pre-therapy CT imaging findings to guide further treatment decisions. Our method mirrored this process. The PAE module simulated the initial phase by evaluating similarities in demographic, pathological, and laboratory test data to construct a graph of patient similarities within a large NSCLC dataset. The results across multiple test datasets confirmed that the PAE achieved a predictive accuracy that was superior to those reported previously [34,35], thus demonstrating its value for identifying similarities among patients with NSCLC undergoing immunotherapy. By integrating radiomics features into the population graph, our DHGN captured both individual- and population-level patterns that were critical for predicting therapeutic benefits. This concurrent integration of multimodal data within a graph network allowed our model to outperform conventional models based solely on either clinical or imaging data, as well as direct composite models [30,36], across multiple survival endpoints. The comparison of the DHGN model and the control models based on clinical characteristics, radiomics, and a combination thereof, confirmed that our two-step approach, leveraging inter-patient similarity via PAE integrated with multimodal data via DHGN, conferred a significant prognostic advantage for immunotherapy outcomes.

Although the DHGN model exhibited only modest differences in C-index across datasets (0.69–0.72), our sensitivity analyses clarified the potential contributors to these variations. Subgroup analyses based on treatment modality and imaging protocol demonstrated that the DHGN model achieved better prognostic performance in patients receiving combination immunotherapy and in those with contrast-enhanced CT scans. These findings are consistent with prior evidence that contrast-enhanced CT captures more tumor-related heterogeneity in the form of radiomic features, and that clinical outcomes vary between combination and monotherapy immunotherapy strategies [37,38]. Through these analyses, this study identified potential factors influencing model performance and further delineated the optimal applicability of the DHGN framework for prognostic modeling in NSCLC immunotherapy. Moreover, the DHGN-response model achieved performance on the MSK dataset comparable to that of the established LORIS method. This highlights the ability of hypergraph networks to match the accuracy of existing benchmarks while remaining operable on standard CPU hardware. With ongoing prospective studies based on the hypergraph model and planned validation using additional multicenter datasets, a hypergraph-based system compatible with routine clinical CPU resources holds the potential to serve as a complement to existing machine learning–based prognostic tools, providing a robust multi–model decision framework for NSCLC immunotherapy.

Compared to the PD-L1 biomarker, our method demonstrated improved utility in guiding immunotherapy decisions with better precision. Although the median PFS among patients with PD-L1 > 49% varied in previous studies, the proportion of such patients in our study (28.9%) is consistent with previous reports [13,39]. The results of our study revealed that DHGN identified an additional 51.3% and 46.7% patients with NSCLC who achieved PFS and OS equivalent to that of patients with PD-L1 > 49%. These findings indicate that our approach enhances the precision of patient selection for immunotherapy compared to current biomarkers. Furthermore, while earlier evidence suggested that patients with lung Sqc may derive greater survival benefits from immunotherapy than those with lung adenocarcinoma [40–42], the results of our study revealed that lower DHGN score patients with lung Sqc had better PFS than those with lung adenocarcinoma, although their OS advantage did not achieve significance. In contrast, higher DHGN score patients with lung Sqc demonstrated significantly improved OS compared with their counterparts with lung adenocarcinoma (*P* = 0.01). This underscores the need for alternative therapeutic regimens in lower DHGN score patients with lung Sqc upon disease progression, to optimize their long-term survival outcomes.

For future clinical translation, our study represents a step toward improving the compatibility of deep learning–based prognostic models with cost-effective clinical computing hardware. Although DHGN requires only 2.50 s per training epoch—substantially faster than EfficientNetV2—the total per-patient processing time increases to 25.50 s when accounting for the extraction of the 1,221 radiomics features. Even so, this represents approximately one-third of the time required by EfficientNetV2. Looking ahead, integrating CPU-compatible hypergraph models into PACS environments, leveraging built-in modules for region-of-interest segmentation and radiomics extraction, may further streamline the workflow. With such integration, DHGN has the potential to markedly enhance the feasibility and efficiency of deploying deep learning tools in routine clinical practice.

Our findings underscore the importance of clinician engagement during model development, as the DHGN demonstrated comparable prognostic performance when trained on available personal computers, in contrast to deep learning networks such as EfficientNetV2, which require GPU-based servers [30]. This enhanced operability and accessibility may facilitate the broader clinician-led implementation of image-based deep learning biomarkers in clinical practices, particularly those without access to high-performance GPU resources.

From a translational perspective, our results highlight three key findings. First, although DHGN was trained on short-term PFS data, it demonstrates clear value in predicting long-term OS in NSCLC patients treated with immunotherapy. Compared with existing radiomics-only, clinical-only, and combined models, DHGN provides superior prognostic performance for OS. In addition, our model successfully identifies low-TMB or low-PD-L1 expression patients who can receive comparable survival benefits from immunotherapy to the high expression patients. Furthermore, the DHGN scores showed a much more monotonic and consistent pattern of PFS and OS than the PD-L1 and TMB expression, leading to more accurate suggestion of patients likely to benefit and more effective exclusion of those unlikely to benefit from immunotherapy. Overall, our results indicate that DHGN could be a reliable tool for improving clinical decision-making practices in precision immunotherapy to maximize patient survival outcomes.

This study had some limitations. First, the external MSK test dataset [25] lacked clinical staging information, thus precluding the inclusion of TNM stage, a key baseline variable, in the PAE module. Future studies should incorporate more extensive test datasets to mitigate potential data biases. Second, not all patients had both PFS and OS endpoint events due to incomplete follow-up data in clinical settings across multiple centers. Improved patient follow-up is needed to further validate the prognostic performance of the DHGN model. In addition, the prognostic comparison between stratified PD-L1 expression and DHGN in the MSK dataset (Table 2) did not achieve the level of significance observed in the other two datasets. One possible explanation for this discrepancy is that patients in the MSK dataset were enrolled prior to 2020 and predominantly received immunotherapy as monotherapy (96.1%), whereas those in the other datasets were mainly enrolled after 2020 and underwent combination immunotherapy (93.3%). Sensitivity analysis revealed the prognostic impact of differences in treatment patterns. Moreover, this finding is consistent with previous observations that combination immunotherapy yielded superior prognostic outcomes relative to monotherapy [39]. Nevertheless, the Kaplan-Meier survival curves presented in Fig 4 show that DHGN offers enhanced prognostic stratification over PD-L1 expression within the MSK cohort. Detailed data relating to this analysis are available on the study’s publicly accessible GitHub repository for further evaluation. Although the PAE used in this study applies a supervised learning strategy to estimate patient similarity, comparison with an unsupervised distance-based clustering approach showed that the supervised method yields better performance for both PFS and OS prediction. Future work should explore semi-supervised and self-supervised strategies to further evaluate how different similarity-learning paradigms influence the DHGN model. Finally, although the TMB cutoff value of 10 mut/Mb used in our study is supported by previously established methodologies, the optimal TMB cutoff values remain controversial [3,33,43]. Future investigations should identify the optimal thresholds for blood-based TMB.

Our two-step DHGN model showed the potential to enhance the prognostic assessment of immunotherapy for NSCLC. The improvements we demonstrated regarding multiple-endpoint survival prediction and patient stratification, alongside the model’s compatibility with standard computing hardware, represent a substantial advancement toward clinician-led personalized cancer care. Future prospective studies with larger and more diverse datasets may further promote the integration of this research paradigm into real-world clinical settings.

## Supporting information

S1 Fig Kaplan-Meier survival curves for the training, ANS test, and MSK test datasets, after stratifying the DHGN model scores using the same proportions as the stratification by tumor mutation burden (41.9:58.1). (DOCX)

S2 Fig Kaplan-Meier survival curves for the high-DHGN and low-DHGN (cutoff: median) patients diagnosed with lung squamous cell carcinoma (Sqc) and adenocarcinoma (Ade), among those in the two test datasets. (DOCX)

S3 Fig In the clinical deployment experiment, ten clinicians evaluated EfficientNetV2 and the DHGN approach developed in this study on a five-point Likert scale. NS: no significant difference. Note: *** represents p < 0.001, and ** represents p < 0.01.
(DOCX)

S4 Fig Schematic of the hypergraph neural network used in the DHGN model. Each patient is represented as a node, and radiomics features are used to construct the hyperedges. All hyperedges are concatenated to generate the hypergraph adjacency matrix. The adjacency matrix and node features are then input into the hypergraph neural network for training, resulting in the final node-level outputs.
(DOCX)

S5 Fig Structure of the hyperedge convolution layer. The HGNN layer follows a node–edge–node transformation. First, the initial node features are processed by a learnable filter matrix to generate refined features. Next, node features are aggregated according to hyperedge membership to form hyperedge features, represented as matrices. Finally, the output node features are obtained by aggregating the corresponding hyperedge features, implemented through multiplication of the hyperedge feature matrices.
(DOCX)

S6 Fig Kaplan–Meier curves (adapted from Figure 3G) truncated at the 90^th^ percentile (A) and 80^th^ percentile (B) of follow-up to improve interpretability. (DOCX)

S7 Fig The calibration plot of DHGN for PFS prediction on the training (A), ANS test (B), and MSK test (C) datasets, respectively. For each dataset, the x-axis shows the model-predicted probability of being progression-free at the cohort-specific median PFS, and the y-axis shows the corresponding Kaplan–Meier estimate of the observed progression-free probability.
(DOCX)

S1 Table C-indexes (with 95% confidence interval) of each model for progression-free survival (PFS) and overall survival (OS) prognosis in the training and two test datasets, with p values representing each model’s comparison to the DHGN. P value was calculated by “*compareC*” function. PFS: progression-free survival, OS: overall survival.
(DOCX)

S2 Table Sensitivity and specificity of PAE models constructed with different numbers of variables, and the corresponding performance of the DHGN model for progression-free survival prediction. *Note:* “n-variable” denotes the PAE model constructed with *n* variables. Reported values represent the average performance across all enumerated models.
(DOCX)

S3 Table C-index of the DHGN models constructed using the corresponding PAE models presented in Table R1. All DHGN results reflect performance for progression-free survival prediction. *Note:* “n-variable” denotes the model built using *n* variables. Reported values represent the average performance across all enumerated models.
(DOCX)

S4 Table Prognostic performance of the DHGN model (constructed using the 9-variable PAE) for predicting progression-free survival and overall survival in patients receiving monotherapy versus combination immunotherapy. Due to the sample size limitations of specific treatment modalities in the MSK dataset, all patients were divided into 70% training and 30% test sets.
(DOCX)

S5 Table Prognostic performance of the DHGN model (constructed using the 9-variable PAE) for predicting progression-free survival and overall survival in patients with non-contrast versus contrast-enhanced CT imaging. Note: P value was not calculated because the patients are different.
(DOCX)

S6 Table Prognostic performance of the DHGN model (constructed using the 9-variable PAE) for predicting progression-free survival and overall survival in first-line versus non–first-line treatment subgroups. (DOCX)
