# Supplementary material for: Clinician-deployable deep hypergraph model integrating clinical and CT radiomics predicts immunotherapy outcomes in NSCLC
Source: PLOS Digit Health. 2026 Apr 20;5(4):e0001361. doi: 10.1371/journal.pdig.0001361 (PMC13095021; doi:10.1371/journal.pdig.0001361)
Supplement: S1 Fig — (DOCX) [file pdig.0001361.s001.docx]

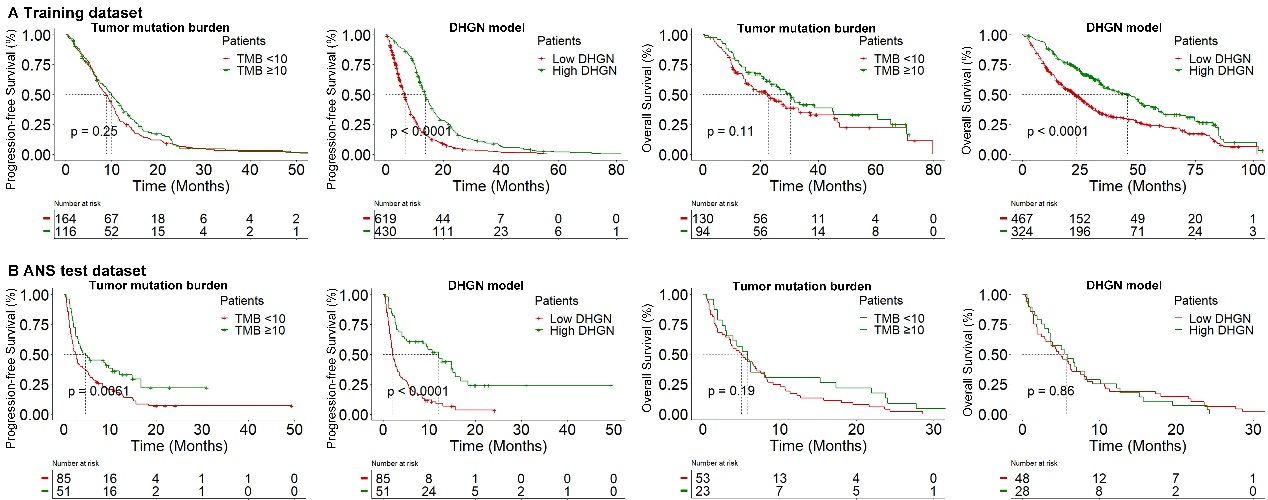


**Figure S1.** Kaplan-Meier survival curves for the training, ANS test, and MSK test datasets, after stratifying the DHGN model scores using the same proportions as the stratification by tumor mutation burden (41.9:58.1).
