# Supplementary material for: Clinician-deployable deep hypergraph model integrating clinical and CT radiomics predicts immunotherapy outcomes in NSCLC
Source: PLOS Digit Health. 2026 Apr 20;5(4):e0001361. doi: 10.1371/journal.pdig.0001361 (PMC13095021; doi:10.1371/journal.pdig.0001361)
Supplement: S2 Fig — (DOCX) [file pdig.0001361.s002.docx]

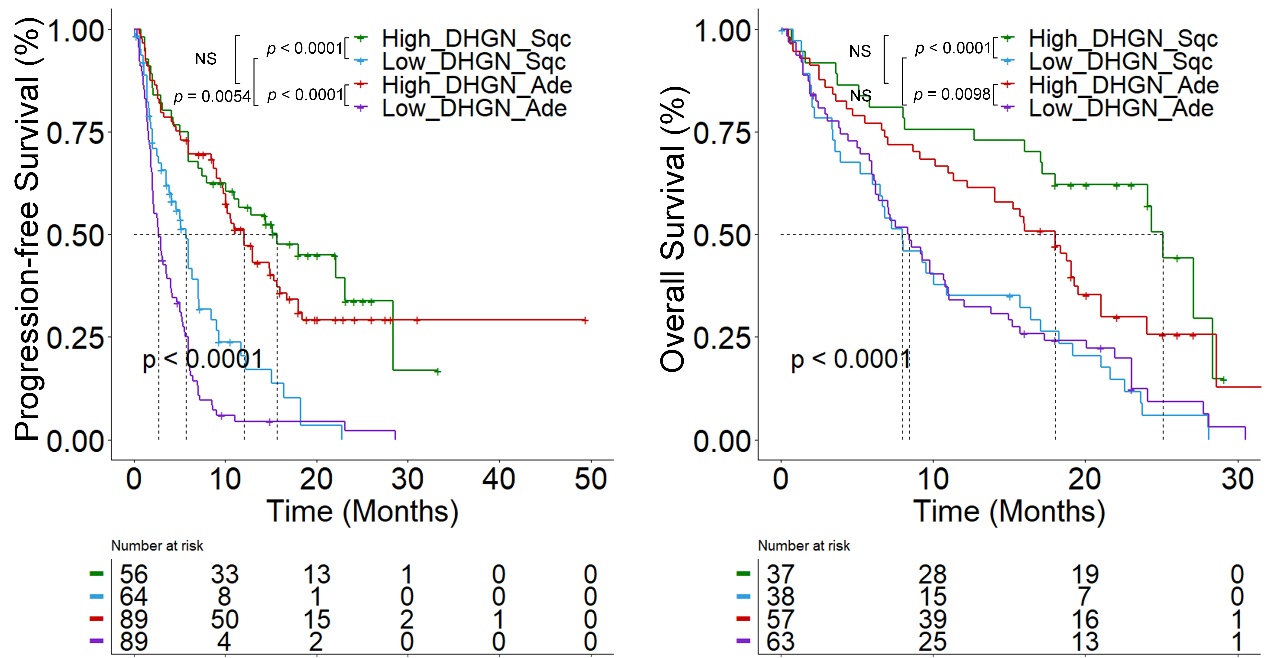


**Figure S2.** Kaplan-Meier survival curves for the high-DHGN and low-DHGN (cutoff: median) patients diagnosed with lung squamous cell carcinoma (Sqc) and adenocarcinoma (Ade), among those in the two test datasets.
