# Supplementary material for: Clinician-deployable deep hypergraph model integrating clinical and CT radiomics predicts immunotherapy outcomes in NSCLC
Source: PLOS Digit Health. 2026 Apr 20;5(4):e0001361. doi: 10.1371/journal.pdig.0001361 (PMC13095021; doi:10.1371/journal.pdig.0001361)
Supplement: S3 Fig — NS: no significant difference. Note: *** represents p < 0.001, and ** represents p < 0.01. (DOCX) [file pdig.0001361.s003.docx]

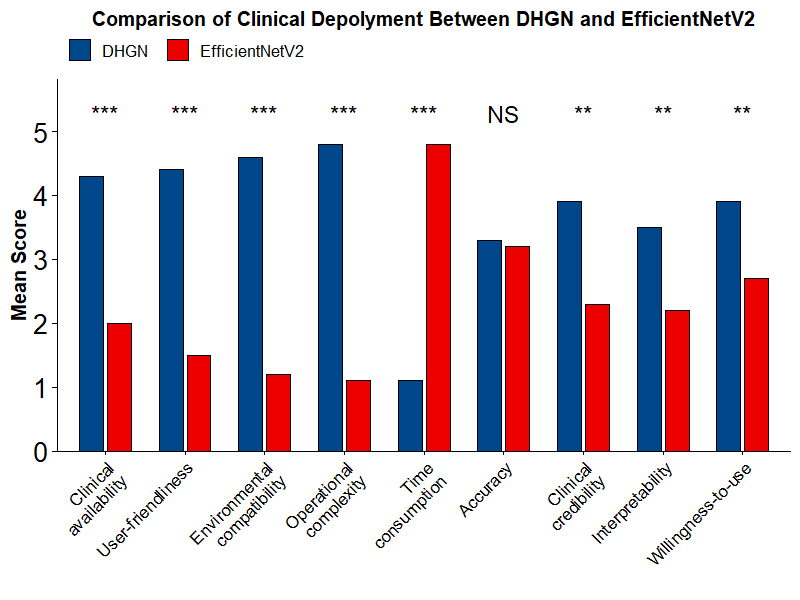


**Figure S3.** In the clinical deployment experiment, ten clinicians evaluated EfficientNetV2 and the DHGN approach developed in this study on a five-point Likert scale. NS: no significant difference. Note: *** represents p<0.001, and ** represents p<0.01.
