# Supplementary material for: Clinician-deployable deep hypergraph model integrating clinical and CT radiomics predicts immunotherapy outcomes in NSCLC
Source: PLOS Digit Health. 2026 Apr 20;5(4):e0001361. doi: 10.1371/journal.pdig.0001361 (PMC13095021; doi:10.1371/journal.pdig.0001361)
Supplement: S4 Fig — Each patient is represented as a node, and radiomics features are used to construct the hyperedges. All hyperedges are concatenated to generate the hypergraph adjacency matrix. The adjacency matrix and node features are then input into the hypergraph neural network for training, resulting in the final node-level outputs. (DOCX) [file pdig.0001361.s004.docx]

**
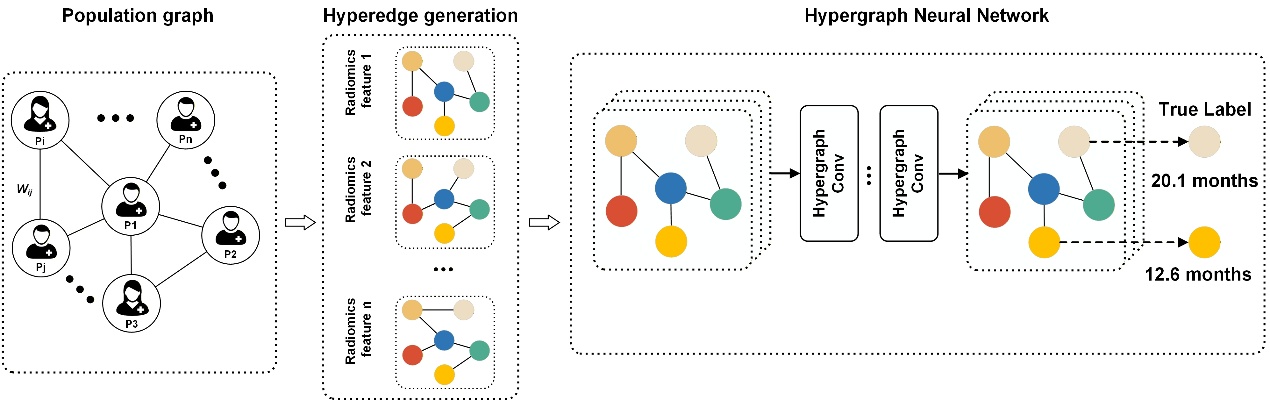
**

**Figure S4.** Schematic of the hypergraph neural network used in the DHGN model. Each patient is represented as a node, and radiomics features are used to construct the hyperedges. All hyperedges are concatenated to generate the hypergraph adjacency matrix. The adjacency matrix and node features are then input into the hypergraph neural network for training, resulting in the final node-level outputs.
