# Supplementary material for: Clinician-deployable deep hypergraph model integrating clinical and CT radiomics predicts immunotherapy outcomes in NSCLC
Source: PLOS Digit Health. 2026 Apr 20;5(4):e0001361. doi: 10.1371/journal.pdig.0001361 (PMC13095021; doi:10.1371/journal.pdig.0001361)
Supplement: S5 Fig — The HGNN layer follows a node–edge–node transformation. First, the initial node features are processed by a learnable filter matrix to generate refined features. Next, node features are aggregated according to hyperedge membership to form hyperedge features, represented as matrices. Finally, the output node features are obtained by aggregating the corresponding hyperedge features, implemented through multiplication of the hyperedge feature matrices. (DOCX) [file pdig.0001361.s005.docx]

**
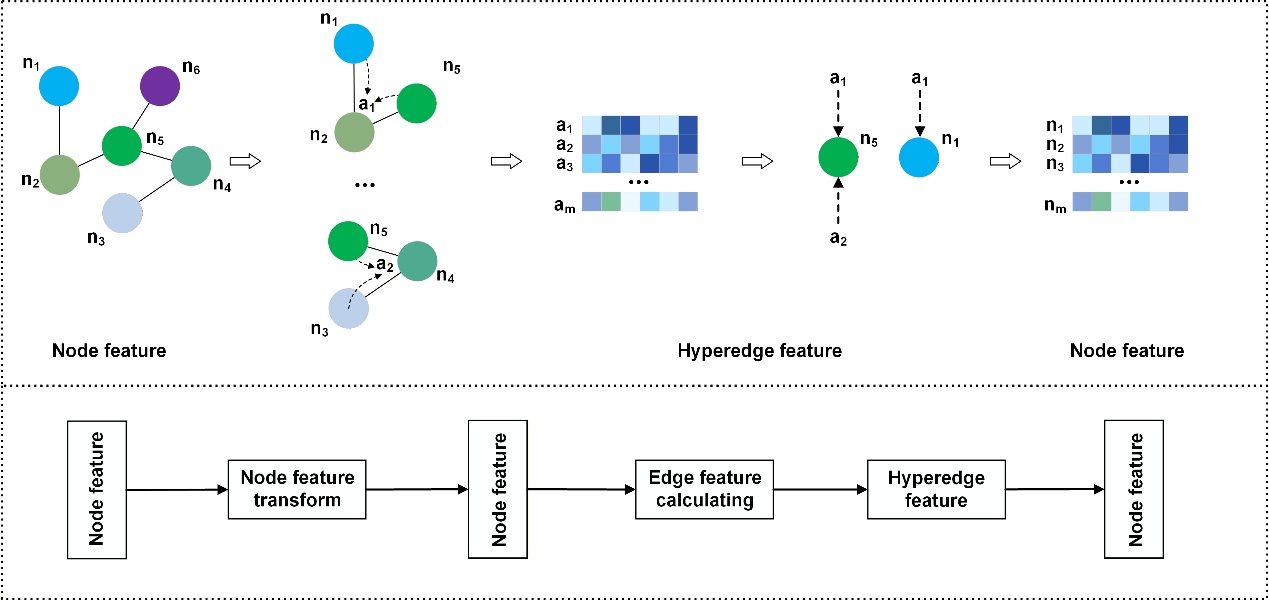
**

**Figure S5.** Structure of the hyperedge convolution layer. The HGNN layer follows a node–edge–node transformation. First, the initial node features are processed by a learnable filter matrix to generate refined features. Next, node features are aggregated according to hyperedge membership to form hyperedge features, represented as matrices. Finally, the output node features are obtained by aggregating the corresponding hyperedge features, implemented through multiplication of the hyperedge feature matrices.
