# Supplementary material for: Clinician-deployable deep hypergraph model integrating clinical and CT radiomics predicts immunotherapy outcomes in NSCLC
Source: PLOS Digit Health. 2026 Apr 20;5(4):e0001361. doi: 10.1371/journal.pdig.0001361 (PMC13095021; doi:10.1371/journal.pdig.0001361)
Supplement: S6 Fig — (DOCX) [file pdig.0001361.s006.docx]

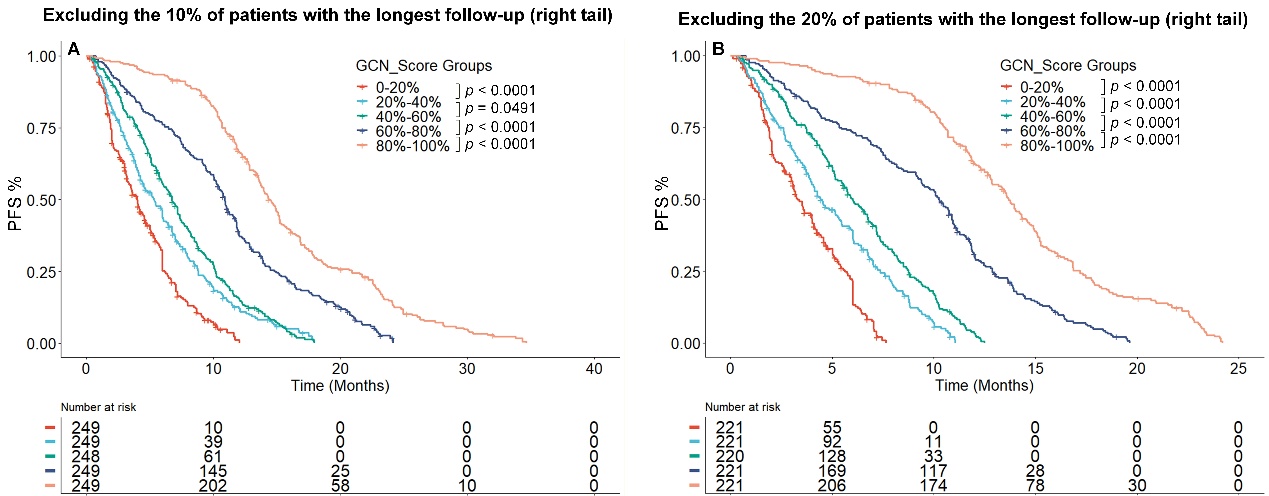


**Figure S6.** Kaplan–Meier curves (adapted from Figure 3G) truncated at the 90^th^ percentile (A) and 80^th^ percentile (B) of follow-up to improve interpretability.
