# Supplementary material for: Clinician-deployable deep hypergraph model integrating clinical and CT radiomics predicts immunotherapy outcomes in NSCLC
Source: PLOS Digit Health. 2026 Apr 20;5(4):e0001361. doi: 10.1371/journal.pdig.0001361 (PMC13095021; doi:10.1371/journal.pdig.0001361)
Supplement: S7 Fig — For each dataset, the x-axis shows the model-predicted probability of being progression-free at the cohort-specific median PFS, and the y-axis shows the corresponding Kaplan–Meier estimate of the observed progression-free probability. (DOCX) [file pdig.0001361.s007.docx]

**
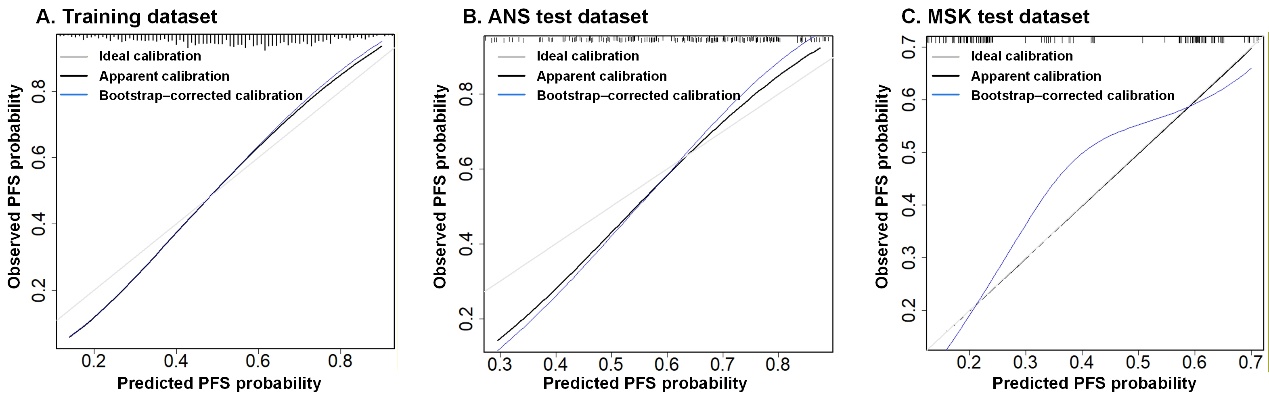
**

**Figure S7.** The calibration plot of DHGN for PFS prediction on the training (A), ANS test (B), and MSK test (C) datasets, respectively. For each dataset, the x-axis shows the model-predicted probability of being progression-free at the cohort-specific median PFS, and the y-axis shows the corresponding Kaplan–Meier estimate of the observed progression-free probability.
