# Supplementary material for: Clinician-deployable deep hypergraph model integrating clinical and CT radiomics predicts immunotherapy outcomes in NSCLC
Source: PLOS Digit Health. 2026 Apr 20;5(4):e0001361. doi: 10.1371/journal.pdig.0001361 (PMC13095021; doi:10.1371/journal.pdig.0001361)
Supplement: S1 Table — P value was calculated by “compareC” function. PFS: progression-free survival, OS: overall survival. (DOCX) [file pdig.0001361.s008.docx]

**Table S1.** C-indexes (with 95% confidence interval) of each model for progression-free survival (PFS) and overall survival (OS) prognosis in the training and two test datasets, with p values representing each model’s comparison to the DHGN. P value was calculated by “*compareC*” function. PFS: progression-free survival, OS: overall survival.

|  | DHGN (Ref) | Radiomics | | Clinical |  | Composite |  |
| --- | --- | --- | --- | --- | --- | --- | --- |
|  | C-indices | C-indices | p | C-indices | p | C-indices | p |
| Training |  |  |  |  |  |  |  |
| PFS | 0.72 (0.68–0.75) | 0.64 (0.62–0.66) | <0.0001 | 0.58 (0.56–0.60) | <0.0001 | 0.63 (0.61–0.65) | <0.0001 |
| OS | 0.70 (0.65–0.75) | 0.61 (0.58–0.64) | 0.003 | 0.52 (0.49–0.54) | 0.0003 | 0.62 (0.59–0.65) | 0.002 |
| ANS Test |  |  |  |  |  |  |  |
| PFS | 0.71 (0.67–0.75) | 0.59 (0.54–0.64) | <0.0001 | 0.55 (0.49–0.60) | <0.0001 | 0.58 (0.53–0.63) | <0.0001 |
| OS | 0.71 (0.66–0.76) | 0.58 (0.52–0.64) | <0.0001 | 0.56 (0.50–0.62) | 0.005 | 0.47 (0.41–0.53) | <0.0001 |
| MSK Test |  |  |  |  |  |  |  |
| PFS | 0.71 (0.66–0.77) | 0.52 (0.46–0.59) | <0.0001 | 0.54 (0.47–0.60) | 0.0004 | 0.54 (0.47–0.60) | 0.0002 |
| OS | 0.69 (0.64–0.73) | 0.55 (0.47–0.62) | 0.005 | 0.43 (0.35–0.50) | 0.005 | 0.40 (0.34–0.49) | 0.007 |
