# Supplementary material for: Clinician-deployable deep hypergraph model integrating clinical and CT radiomics predicts immunotherapy outcomes in NSCLC
Source: PLOS Digit Health. 2026 Apr 20;5(4):e0001361. doi: 10.1371/journal.pdig.0001361 (PMC13095021; doi:10.1371/journal.pdig.0001361)
Supplement: S2 Table — Note: “n-variable” denotes the PAE model constructed with n variables. Reported values represent the average performance across all enumerated models. (DOCX) [file pdig.0001361.s009.docx]

**Table S2.** Sensitivity and specificity of PAE models constructed with different numbers of variables, and the corresponding performance of the DHGN model for progression-free survival prediction. *Note:* “n-variable” denotes the PAE model constructed with *n* variables. Reported values represent the average performance across all enumerated models.

|  | Training dataset | | | ANS test dataset | | | MSK test dataset | | |
| --- | --- | --- | --- | --- | --- | --- | --- | --- | --- |
|  | Sensitivity | Specificity | P | Sensitivity | Specificity | P | Sensitivity | Specificity | P |
| 3-variable | 68.9% | 63.1% | Ref | 62.1% | 60.3% | Ref | 61.1% | 59.3% | Ref |
| 5-variable | 72.3% | 68.6% | 0.101 | 67.3% | 65.6% | 0.063 | 63.6% | 66.7% | 0.137 |
| 7-variable | 85.6% | 87.6% | 0.021 | 86.3% | 85.4% | 0.002 | 81.7% | 82.3% | 0.018 |
| 9-variable | 91.3% | 95.1% | <0.001 | 93.3% | 89.5% | <0.001 | 91.6% | 93.8% | <0.001 |
