# Supplementary material for: Clinician-deployable deep hypergraph model integrating clinical and CT radiomics predicts immunotherapy outcomes in NSCLC
Source: PLOS Digit Health. 2026 Apr 20;5(4):e0001361. doi: 10.1371/journal.pdig.0001361 (PMC13095021; doi:10.1371/journal.pdig.0001361)
Supplement: S3 Table — All DHGN results reflect performance for progression-free survival prediction. Note: “n-variable” denotes the model built using n variables. Reported values represent the average performance across all enumerated models. (DOCX) [file pdig.0001361.s010.docx]

**Table S3.** C-index of the DHGN models constructed using the corresponding PAE models presented in Table R1. All DHGN results reflect performance for progression-free survival prediction. *Note:* “n-variable” denotes the model built using *n* variables. Reported values represent the average performance across all enumerated models.

|  | Training dataset | | | ANS test dataset | | | MSK test dataset | | |
| --- | --- | --- | --- | --- | --- | --- | --- | --- | --- |
|  | C-index | 95% CI | P | C-index | 95% CI | P | C-index | 95% CI | P |
| 3-variable | 0.55 | (0.50-0.60) | Ref | 0.53 | (0.50-0.58) | Ref | 0.51 | (0.45-0.56) | Ref |
| 5-variable | 0.61 | (0.54-0.68) | 0.073 | 0.59 | (0.51-0.67) | 0.050 | 0.55 | (0.50-0.60) | 0.530 |
| 7-variable | 0.68 | (0.63-0.73) | <0.001 | 0.64 | (0.60-0.68) | 0.005 | 0.63 | (0.59-0.68) | 0.009 |
| 9-variable | 0.72 | (0.68–0.75) | <0.001 | 0.71 | (0.67–0.75) | <0.001 | 0.71 | (0.66–0.77) | <0.001 |
