# Supplementary material for: Clinician-deployable deep hypergraph model integrating clinical and CT radiomics predicts immunotherapy outcomes in NSCLC
Source: PLOS Digit Health. 2026 Apr 20;5(4):e0001361. doi: 10.1371/journal.pdig.0001361 (PMC13095021; doi:10.1371/journal.pdig.0001361)
Supplement: S4 Table — Due to the sample size limitations of specific treatment modalities in the MSK dataset, all patients were divided into 70% training and 30% test sets. (DOCX) [file pdig.0001361.s011.docx]

**Table S4.** Prognostic performance of the DHGN model (constructed using the 9-variable PAE) for predicting progression-free survival and overall survival in patients receiving monotherapy versus combination immunotherapy. Due to the sample size limitations of specific treatment modalities in the MSK dataset, all patients were divided into 70% training and 30% test sets.

|  | Progression-free survival | | Overall survival | |
| --- | --- | --- | --- | --- |
|  | C-index | 95% CI | C-index | 95% CI |
| Monotherapy |  |  |  |  |
| Train dataset | 0.69 | 0.64-0.74 | 0.65 | 0.63-0.68 |
| Test dataset | 0.67 | 0.63-0.71 | 0.64 | 0.61-0.67 |
| Combined therapy |  |  |  |  |
| Train dataset | 0.75 | 0.70-0.80 | 0.71 | 0.61-0.81 |
| Test dataset | 0.72 | 0.63-0.81 | 0.70 | 0.65-0.75 |
