# Supplementary material for: Clinician-deployable deep hypergraph model integrating clinical and CT radiomics predicts immunotherapy outcomes in NSCLC
Source: PLOS Digit Health. 2026 Apr 20;5(4):e0001361. doi: 10.1371/journal.pdig.0001361 (PMC13095021; doi:10.1371/journal.pdig.0001361)
Supplement: S5 Table — Note: P value was not calculated because the patients are different. (DOCX) [file pdig.0001361.s012.docx]

**Table S5.** Prognostic performance of the DHGN model (constructed using the 9-variable PAE) for predicting progression-free survival and overall survival in patients with non-contrast versus contrast-enhanced CT imaging. Note: P value was not calculated because the patients are different.

|  | Progression-free survival | | Overall survival | |
| --- | --- | --- | --- | --- |
|  | C-index | 95% CI | C-index | 95% CI |
| non-contrast CT |  |  |  |  |
| Train dataset | 0.65 | 0.60-0.70 | 0.60 | 0.53-0.67 |
| Test dataset | 0.62 | 0.55-0.69 | 0.58 | 0.52-0.64 |
| contrast-enhanced CT |  |  |  |  |
| Train dataset | 0.77 | 0.72-0.82 | 0.72 | 0.65-0.79 |
| Test dataset | 0.73 | 0.61-0.85 | 0.71 | 0.67-0.75 |
