# Supplementary material for: Clinician-deployable deep hypergraph model integrating clinical and CT radiomics predicts immunotherapy outcomes in NSCLC
Source: PLOS Digit Health. 2026 Apr 20;5(4):e0001361. doi: 10.1371/journal.pdig.0001361 (PMC13095021; doi:10.1371/journal.pdig.0001361)
Supplement: S6 Table — (DOCX) [file pdig.0001361.s013.docx]

**Table S6.** Prognostic performance of the DHGN model (constructed using the 9-variable PAE) for predicting progression-free survival and overall survival in first-line versus non–first-line treatment subgroups.

|  | Progression-free survival | | Overall survival | |
| --- | --- | --- | --- | --- |
|  | C-index | 95% CI | C-index | 95% CI |
| First-line therapy |  |  |  |  |
| Train dataset | 0.72 | 0.65-0.79 | 0.68 | 0.63-0.73 |
| Test dataset | 0.71 | 0.66-0.77 | 0.69 | 0.66-0.73 |
| Non–first-line |  |  |  |  |
| Train dataset | 0.70 | 0.66-0.75 | 0.68 | 0.62-0.74 |
| Test dataset | 0.69 | 0.63-0.74 | 0.68 | 0.65-0.71 |
